# Symptom burden, palliative care need and predictors of physical and psychological discomfort in two UK hospitals

**DOI:** 10.1186/1472-684X-12-11

**Published:** 2013-02-26

**Authors:** Tony Ryan, Christine Ingleton, Clare Gardiner, Chris Parker, Merryn Gott, Bill Noble

**Affiliations:** 1 School of Nursing & Midwifery, University of Sheffield, Barber House, S10 2HQ, Sheffield, UK; 2 School of Health and Related Research, University of Sheffield, Regent Court, 30 Regent Street, S1 4DA, Sheffield, UK; 3 Faculty of Medical and Health Sciences, University of Auckland, 1142, Auckland, New Zealand; 4 Academic Unit of Supportive Care, University of Sheffield, Barber House, S10 2HQ, Sheffield, UK

**Keywords:** Palliative care, Burden of illness, Psychological stress, Prevalence, Dementia, End of life care, Supportive care

## Abstract

**Background:**

The requirement to meet the palliative needs of acute hospital populations has grown in recent years. With increasing numbers of frail older people needing hospital care as a result of both malignant and non-malignant conditions, emphasis is being placed upon understanding the physical, psychological and social burdens experienced by patients. This study explores the extent of burden in two large UK hospitals, focusing upon those patients who meet palliative care criteria. Furthermore, the paper explores the use of palliative services and identifies the most significant clinical diagnostic and demographic factors which determine physical and psychological burden.

**Methods:**

Two hospital surveys were undertaken to identify burden using the Sheffield Profile for Assessment and Referral to Care (SPARC). The Gold Standards Framework (GSF) is used to identify those patients meeting palliative care criteria. Participants were identified as being in-patients during a two-week data collection phase for each site. Data was gathered using face-to-face interviews or self-completion by patients or a proxy. Descriptive analyses highlight prevalence and use of palliative care provision. Binary logistic regression assesses clinical diagnostic predictor variables of physical and psychological burden.

**Results:**

The sample consisted of 514 patients and elevated physical, psychological and social burden is identified amongst those meeting palliative care criteria (n = 185). Tiredness (34.6%), pain (31.1%), weakness (28.8%) and psychological discomfort (low mood 19.9%; anxiety 16.1%) are noted as being prevalent. A small number of these participants accessed Specialist Palliative Care (8.2%). Dementia was identified as a predictor of physical (OR 3.94; p < .05) and psychological burden (OR 2.88; p < .05), being female was a predictor of psychological burden (OR 2.00; p < .05).

**Conclusion:**

The paper highlights elevated levels of burden experienced by patients with palliative care requirements. Moreover, the paper also indicates that a large proportion of such patients are not in receipt of palliative approaches to their care. Furthermore, the paper identifies that those with non-malignant illnesses, especially dementia, may experience high levels of physical and psychological burden.

## Background

The number of people in the UK dying in a secondary care facility is set to rise by 20 per cent in the coming decades [1]. This trend has not been lost on UK policy makers with the End of Life Care Strategy for England placing emphasis on the development of capacity to provide high quality palliative and supportive care for all patients, in all settings, including hospitals [2]. Policy development of this nature is occurring against a backdrop of global ageing, a rising prevalence of frailty and co-morbidity in chronic illness [3]. Meeting the palliative needs of such diverse populations is challenging and is made more difficult with growing evidence that patients face significant physical and psychological burden [4]. Elevated prevalence of psychological and physical burdens towards the end of life have been noted in patients with cancer [5], chronic kidney disease [6], COPD [7] and heart failure [8]. Sources of physical symptoms are broad and have multiple aetiologies. Cancer pain can occur as a result of tumour pervasion or pharmacological toxicity. Physiological change also contributes to physical pain for patients with COPD [9] and heart failure [10]. Psychological burden is often triggered as a result of rumination [11,12]. The root causes of anxiety at the end of life can be attributed to adverse drug effects, changes in metabolism, existential concerns in relation to death and loss as well as in response to chronic pain [13]. Other work has noted the interaction between physical, psychological and social aspects of chronic illness at the end of life and its impact upon burden. Experiential accounts reveal that physical and psychological burden are difficult to separate from one-another [14]. The relationship between physical and psychological burden is noted within the literature on dyspnea and the notion of ‘total dyspnea’ as a result of the association between physical burden, psychological, spiritual and social distress [15]. Evidence of the co-existence of pain, depression and fatigue in advanced cancer also exists [16]. Although we are beginning to understand the prevalence of palliative care needs within hospital sites [17] we know little about the specific burden associated with these patients across hospital populations. We do know that very few patients access Specialist Palliative Care (SPC) in hospital settings and we also suspect that this is not indicative of need [17-19] and of those patients with palliative care needs little is known about which diagnostic and demographic factors might contribute to physical and psychological burden. This latter point being of particular concern to those seeking to develop palliative care services for patients suffering from non-malignant diseases.

This paper sets out to achieve three aims. Firstly, it will identify symptom burden across a sample of hospitalised patients. Secondly, it will identify the proportion of those patients who have palliative care need, comparing the prevalence of burden between palliative and non-palliative patients. Thirdly, the paper also seeks to identify which clinical diagnostic groups predict high levels of physical and psychological burden and the degree of SPC service utilisation within the sub-population meeting palliative care criteria. The paper is derived from data collected through two large scale hospital surveys seeking to establish the palliative care needs of such populations in the UK. As far as the authors are aware, this is the first study of its kind in that it includes an assessment of patients who have not been referred to SPC services as well as those who have.

## Method

A prospective survey (census) of in-patients was undertaken in two English hospitals selected for socio-demographic diversity. The Sheffield Northern General Hospital (SNGH) serves a largely urban, economically disadvantaged and ethnically diverse area; in contrast the Royal Lancaster Infirmary (RLI) serves a predominantly white Caucasian semi-rural/remote rural population.

The survey of SNGH was undertaken over an 11 day period in May 2010 and the survey of RLI over a five day period in November 2010. All in-patient wards except children’s wards and mother and baby units were included. Each ward was visited by two members of the data collection team at some point during the survey period, all patients aged 18 years and over and resident on the ward on the morning of that day were eligible for inclusion. Due to a lack of translation facilities non-English speaking patients were excluded. Senior medical and nursing staff, and relatives or close friends (where available) were consulted to identify any patients lacking capacity to consent. Face-to-face interview and self-completion was used to administer the survey, based upon participant preference. The approach to the inclusion of patients lacking capacity was developed in line with the Mental Capacity Act and related guidance [20]. For these patients, a personal consultee (relative or close friend) was approached and invited to participate. A fuller description of the methods employed within this study is reported elsewhere [18]. The following data were collected for patients/consultees who consented to participation:

1. Collection of data from patients’ hospital case notes comprising: evidence of palliative care need according to Gold Standards Framework (GSF) prognostic indicator criteria [21].

2. Data relating to the burden of physical and psychological symptoms using the Sheffield Profile for Assessment and Referral to Care index (SPARC). SPARC is a validated screening tool used to identify distressing symptoms caused by an advanced illness [22]. The tool concentrates on the degree of burden in the past month for a number of specific symptoms in seven separate sub-scales: Physical burden; psychological burden; religious and spiritual issues; independence; family and social; treatment; personal affairs.

All data were collected by a team of 30 experienced researchers. Data collected from hospital case notes was undertaken by researchers with a clinical background in medicine or nursing. All researchers undertook training in survey methodology and data collection prior to the study commencing. All data were entered into SPSS v16 and data cleaning was undertaken. Descriptive analyses were performed in order to characterise the sample, identify the range and prevalence of symptoms and depict utilisation of palliative care services. The GSF prognostic indicator guide provides 11 diagnostic criteria categories which give an indication of patients who might benefit from palliative care input. Patients meeting these criteria were identified (GSF group). Statistical testing on the differences between the GSF group and non-GSF group were not undertaken as sample size and recruitment were not devised with these comparisons in mind. With a policy emphasis placed upon the development of a workforce able to provide support in meeting the psychological and physical needs analysis also focused upon these aspects of SPARC and, in particular, set out to determine those factors which predict burden within these subscales. SPARC guidance recommends referral to a medical practitioner should a patient report being ‘very much distressed’ to any of the items within the assessment schedule. In practice this warrants immediate further investigation and potential treatment. We have used this as the basis of an indication of burden here. Further to this binary logistic regression was performed to assess whether known diagnostic (GSF indicator; number of co-morbidities) and/or demographic factors (age; sex; living arrangements) were able to predict physical or psychological burden. Each predictor variable was entered singly and not adjusted for other variables. Mode of completion, proxy or otherwise, was also entered as a factor as it was considered that this may have a bearing on reported burden. Ethical approval for the study was granted by Nottingham 1 Research Ethics Committee. Research Governance approval was granted by the relevant NHS Trusts.

## Results

A total of 1359 in-patients were eligible for inclusion in the survey (1009 patients in Sheffield and 350 patients in Lancaster). Of the total eligible patient population, 654 patients agreed to participate; 616 patients consented for themselves and 38 consented via a consultee. Over half of these (55.9 per cent) were researcher administered via face-to-face interviews. Response rates were similar for the two hospitals (SNGH 46.9%, RLI 52.9%). Complete data sets were available for 514 patients in the study.

Of the 514 patients in the sample, just over one-third (n = 185, 36.2%) met one or more of these 11 diagnostic criteria for palliative care need. These 185 patients are included in descriptive analysis presented below. After data cleaning and removal of two cases with substantial missing data relating to clinical diagnosis, 183 patients were included in binary regression analyses aimed at identifying the diagnostic factors predicting symptoms.

The mean age of participating patients was 68 (SD 19.1). The mean age of those meeting GSF criteria and included in the analysis here was 73.7 years (SD 15.4). Those meeting GSF criteria were significantly older than the non-GSF participants in the sample (mean difference 7.2 years (95% CI 3.9-10.5)). GSF and non-GSF were similar in terms of gender; 96 were female (52.7 per cent) compared with 52.7 per cent of the non-GSF sample (n = 271). Almost half (45.4 per cent, n = 83) lived with other people, such as a spouse, just over two fifths (38.3 per cent, n = 70) lived alone in the community, with the remainder (9.8 per cent, n = 18) (12 missing) having been admitted to hospital from a residential or nursing home. This was different to those not meeting GSF criteria where a larger proportion lived with another person (58.6 per cent, n = 191) and a smaller proportion had been admitted from a residential or nursing home (1.5 per cent, n = 5). A similar proportion lived alone in the community (39.9 per cent, n = 130).

Tables 1, 2 and 3 summarise the data from patient/consultee completed questionnaires, concerning physical, psychological, religious/spiritual, independence, family/social and treatment sub-scales for all study participants with a full data set (n = 514). Each table presents data for those participants meeting GSF criteria (GSF Group) and for those patients not meeting GSF criteria (non-GSF group). The responses were made in answer to the question “have you had worrying thoughts or distress caused in the past month by....” followed by each item. Considerable burden is noted across all six subscales, particularly within those associated with physical, psychological and independence components of the SPARC tool. The three most prevalent physical symptoms that patients found that they were ‘very much distressed’ by were: tiredness; pain and weakness. The three most prevalent psychological symptoms that patients found that they were ‘very much distressed’ by were: feelings of low mood; feeling anxious and the feeling that everything is an effort. It is notable that the independence sub-scale also demonstrated a high prevalence of burden, particularly in relation to losing independence and changes in ability to carry out household and personal care tasks. These tables also reveal elevated burden amongst the GSF group of participants when compared with non-GSF patients for a number of items in the physical sub-scale (Table 1), psychological sub-scale (Table 2) as well as independence, religious and spirituality sub-scales (Table 3).

**Table 1:** Prevalence of burden within physical sub-scales by GSF or non-GSF criteria

**Physical symptoms**	**GSF group**				**Non-GSF group**				
**Subscale and item**	**Very much**	**Quite a bit**	**A little bit**	**Not at all**	**Very much**	**Quite a bit**	**A little bit**	**Not at all**	**Missing**
	**n(%)**	**n(%)**	**n(%)**	**n(%)**	**n(%)**	**n(%)**	**n(%)**	**n(%)**	**n(%)**
Distressed or bothered by pain	56 (31.1)	37 (20.6)	38 (21.1)	49 (27.2)	114 (36.2)	84 (26.7)	57 (18.1)	60 (19)	19 (3.7)
Loss of memory	25 (13.9)	17 (9.4)	45 (25)	93 (51.3)	15 (4.9)	19 (6.2)	72 (23.4)	202 (65.6)	26 (5.1)
Headache	7 (3.9)	14 (7.8)	45 (25.1)	113 (63.1)	16 (5.2)	37 (12)	60 (19.5)	195 (63.3)	27 (5.3)
Dry mouth	31 (17.1)	56 (30.9)	51 (28.2)	43 (23.8)	56 (17.9)	53 (17)	88 (28.2)	115 (36.9)	21 (4.1)
Sore mouth	9 (5)	16 (8.8)	29 (16)	127 (70.2)	10 (3.3)	19 (6.3)	41 (13.5)	233 (76.9)	30 (5.8)
Shortness of breath	42 (23.1)	41(22.5)	43 (23.6)	56 (30.8)	42 (13.3)	36 (11.4)	71 (22.5)	166 (52.7)	17 (3.3)
Cough	19 (10.5)	37 (20.4)	39 (21.5)	86 (47.5)	15 (4.9)	34 (11.1)	81 (26.5)	176 (57.5)	27 (5.3)
Feeling sick (nausea)	16 (8.8)	24 (13.3)	40 (22.1)	101 (55.8)	30 (9.6)	41 (13.2)	73 (23.5)	167 (53.7)	22 (4.3)
Being sick (vomiting)	11 (6.1)	14 (7.8)	24 (13.4)	130 (72.6)	29 (9.4)	21 (6.8)	43 (14)	214 (69.7)	28 (5.4)
Bowel problems	35 (19.2)	35 (19.2)	32 (17.6)	80 (44)	48 (15)	48 (15)	66 (20.7)	157 (49.2)	13 (2.5)
Bladder problems	24 (13.3)	21 (11.6)	25 (13.8)	111 (61.3)	31 (10)	31 (10	47 (15.1)	202 (65)	22 (4.3)
Feeling weak	53 (28.8)	54 (29.3)	40 (21.7)	37 (20.1)	40 (12.7)	85 (27.1)	86 (27.4)	103 (32.8)	16 (3.1)
Feeling tired	63 (34.6)	57 (31.3)	43 (23.6)	19 (10.4)	85 (26.7)	92 (28.9)	76 (23.9)	65 (20.4)	14 (2.7)
Problems sleeping at night	34 (18.9)	31 (17.2)	33 (18.3)	82 (45.6)	73 (23.2)	70 (22.2)	51 (16.2)	121 (38.4)	19 (3.7)
Feeling sleepy during the day	33 (17.9)	69 (37.5)	54 (29.3)	28 (15.2)	56 (17.7)	77 (24.4)	112 (35.4)	71 (22.5)	14 (2.7)
Loss of appetite	33 (18)	34 (18.6)	45 (24.6)	71 (38.8)	45 (14.2)	59 (18.6)	67 (21.1)	147 (46.2)	13 (2.5)
Changes in your weight	32 (17.7)	41(22.7)	40 (22.1)	68 (37.6)	37 (11.9)	38 (12.2)	77 (24.7)	160 (51.3)	21 (4.1)
Swallowing	12 (6.7)	6 (3.4)	25 (13.4)	135 (75.8)	12 (3.8)	12 (3.8)	29 (9.2)	261 (83.1)	22 (4.3)
Changes in your appearance	13 (7.2)	24 (13.3)	25 (13.9)	118 (65.6)	10 (3.2)	22 (7)	41 (13.1)	240 (76.7)	21 (4.1)
Restless and agitated	22 (12.2)	34 (18.9)	55 (30.6)	69 (38.3)	28 (8.8)	47 (14.8)	76 (23.9)	167 (52.5)	16 (3.1)
Symptoms not being under control	19 (10.4)	29 (15.8)	46 (25.1)	81 (44.3)	34 (10.8)	26 (8.2)	72 (22.8)	184 (58.2)	30 (5.8)

**Table 2:** Prevalence of burden within psychological sub-scales by GSF or non-GSF criteria

**Psychological symptoms**	**GSF group**				**Non-GSF group**				
**Subscale and item**	**Very Much**	**Quite a bit**	**A little bit**	**Not at all**	**Very Much**	**Quite a bit**	**A little bit**	**Not at all**	**Missing**
	**n(%)**	**n(%)**	**n(%)**	**n(%)**	**n(%)**	**n(%)**	**n(%)**	**n(%)**	**n(%)**
Anxiety	29 (16.1)	49 (27.2)	48 (26.7)	54 (30)	32 (10)	54 (16.9)	94 (29.5)	139 (43.6)	15 (2.9)
Low mood	36 (19.9)	43 (23.8)	55 (30.4)	47 (26)	34 (10.7)	50 (15.8)	100 (31.5)	133 (41.9)	16 (3.1)
Confusion	26 (14.4)	12 (6.6)	42 (23.2)	101 (55.8)	16 (5.1)	19 (6.1)	50 (16)	228 (72.8)	20 (3.9)
Unable to concentrate	26 (14.4)	28 (15.6)	43 (23.9)	83 (46.1)	20 (6.3)	36 (11.3)	80 (25.2)	182 (57.2)	16 (3.1)
Loneliness	27 (14.8)	34 (18.7)	44 (24.2)	77 (42.3)	28 (8.9)	31 (9.9)	43 (13.7)	211 (67.4)	19 (3.7)
Everything is an effort	29 (16.3)	46 (25.8)	55 (30.9)	48 (27)	41 (13)	46 (14.6)	91 (28.8)	138 (43.7)	20 (3.9)
Life is not worth living	16 (8.9)	24 (13.4)	33 (18.4)	106 (59.2)	18 (5.9)	18 (5.9)	33 (10.8)	237 (77.5)	29 (5.6)
Thoughts about ending it all	8 (4.5)	13 (7.3)	17 (9.6)	139 (78.5)	14 (4.6)	7 (2.3)	18 (5.9)	268 (87.3)	30 (5.8)
Effects on your sexual life	11 (7.6)	11 (7.6)	18 (12.4)	105 (72.4)	17 (6.3)	13 (4.8)	28 (10.3)	213 (78.6)	98 (19.1)

**Table 3:** Prevalence of burden within religious & spiritual, independence, family and social and treatment sub-scales by GSF or non-GSF criteria

	**GSF group**				**Non-GSF group**				
**Subscale and item**	**Very much**	**Quite a bit**	**A little bit**	**Not at all**	**Very much**	**Quite a bit**	**A little bit**	**Not at all**	**Missing**
	**n(%)**	**n(%)**	**n(%)**	**n(%)**	**n(%)**	**n(%)**	**n(%)**	**n(%)**	**n(%)**
**Religious & Spiritual**									
Thoughts about death or dying.	9 (5.1)	11 (6.2)	32 (18.1)	125(70.6)	15 (4.8)	17 (5.4)	42 (13.5)	238 (76.3)	25 (4.9)
Religious or spiritual needs not being met.	4 (2.2)	5 (2.8)	13 (7.3)	157 (87.7)	4 (1.3)	4 (1.3)	13 (4.2)	287 (93.2)	27 (5.3)
**Independence**									
Losing your independence.	36(20.1)	39(21.8)	38 (21.2)	66 (36.9)	49 (15.4)	29 (9.1)	69 (21.6)	172 (53.9)	16 (3.1)
Changes in your ability to carry out usual activities.	32(17.6)	42 (23.1)	42(23.1)	66 (36.3)	40 (12.7)	49 (15.6)	62 (19.7)	163 (51.9)	18 (3.5)
Changes in your ability to carry out usual household tasks.	36 (20.1)	37 (20.7)	34 (19)	72 (40.2)	38 (12.1)	50 (16)	58 (18.5)	167 (53.4)	22 (4.3)
**Family & Social**									
Feeling that people do not understand what you want.	7 (3.8)	33 (18.1)	38 (20.9)	104 (57.1)	20 (6.3)	24 (7.6)	47 (14.9)	225 (71.2)	16 (3.1)
The effect that your illness is having on your family and other people.	34 (18.7)	55 (30.2)	42 (23.1)	51 (28)	49 (15.3)	63 (19.6)	98 (30.5)	111 (34.6)	11 (2.1)
Lack of support from your family and other people.	6(3.3)	7 (3.9)	18 (9.9)	150 (82.9)	9 (2.8)	7 (2.2)	15 (4.7)	286 (90.2)	16 (3.1)
Needing more help than your family can give.	11 (6.3)	17 (9.7)	25 (14.3)	122 (69.7)	14 (4.5)	27 (8.6)	39 (12.4)	234 (74.5)	25 (4.9)
**Treatment**									
Side effects from your treatments.	11 (6.2)	22 (12.4)	38 (21.5)	106 (59.9)	17 (5.4)	31 (9.9)	54 (17.3)	211 (67.4)	24 (4.7)
The long term effects of your treatment.	22 (12.4)	22 (12.4)	37 (20.8)	97 (54.5)	34 (10.8)	26 (8.2)	72 (22.8)	184 (58.2)	20 (3.9)

Data on the extent of a palliative or supportive care approach is only available for those patients in the study who met with GSF criteria and for whom a full data set is available (n = 183). Of these patients just 15 had been referred to SPC services (8.2 per cent), 53 (28 per cent) had a Do Not Attempt to Resuscitation Order in place, two (1.1 per cent) had been placed on the Liverpool Care Pathway (LCP) and nine (4.9 per cent) were prescribed long term opiates. Of the 15 patients referred to SPC services, 11 had a primary diagnosis of cancer.

The GSF data were explored by looking at a range of factors which might contribute to likelihood of such physical and psychological burden for the GSF group. Table 4 summarises the proportion of patients experiencing burden according to the ‘very much distressed’ criteria within each GSF prognostic indicator group. It should be noted that patients with dementia were more likely than any other group to be distressed by at least one physical symptom and at least one psychological symptom.

**Table 4:** Proportion of participants expressing significant distress in at least one item of psychological or physical burden

**GSF prognostic indicator criteria**	**Proportion of disease condition with psychological burden**	**Proportion of disease condition with physical burden**
	**N (%)**	**N (%)**
GSF Cancer	13 (39)	25 (78)
GSF Heart disease	15 (39)	23 (60)
GSF COPD	18 (52)	27 (81)
GSF Renal disease	4 (26)	11 (73)
GSF Frailty	26 (53)	34 (70)
GSF Dementia	21 (65)	29 (90)
GSF Stroke	10 (58)	15 (88)
GSF Other	12 (42)	19 (70)

The results of the binary logistic regression analyses are presented in Table 5 The results indicate that patients diagnosed with heart disease were least likely to be very distressed by physical symptoms (OR 0.42). The results also indicate that patients with dementia were more likely to be very distressed by physical symptoms (OR 3.94) and very distressed by psychological symptoms (OR 2.88). Being female (OR 2.00) was a predictor of psychological symptom burden. Age was not a predictor of either physical or psychological burden (OR 1.00). It is noted above that the authors were cognisant of the effect of proxy reporting on the nature of patient burden. Just over one in ten of those meeting GSF criteria (12.6 per cent, n = 23) had a proxy informant. Of these, 20 reported on behalf of patients meeting GSF dementia criteria (60.6 per cent). Adding proxy informant as an indicator in the model diminishes the effect of dementia as a predictor of psychological burden (OR 1.11 (95% CI 0.38, 3.28) p = 0.159), whilst the presence of a proxy indicator becomes significant (OR 6.41 (95% CI 1.57, 26.07) p = 0.009). Similarly the effect of dementia as a predictor of physical burden is also diminished when adding the proxy informant variable (OR 2.98 (95% CI 0.65, 13.55) p = 0.159). In the latter case proxy informant as an indicator is not, however, a predictor (OR 1.72 (95% CI 0.27, 10.79).

**Table 5:** The results of binary logistic regression analysis to predict psychological and physical burden

	**Physical burden**			**Psychological burden**		
**Predictor**	**Odds ratio**	**95% CI**	**Sig. (p)**	**Odds ratio**	**95% CI**	**Sig.**
GSF cancer	1.27	(0.51, 3.17)	0.611	0.78	(0.36, 1.68)	0.519
GSF heart	**0.42**	**(0.20, 0.91)**	**0.027**	0.77	(0.37, 1.60)	0.488
GSF COPD	1.67	(0.64, 4.36)	0.293	1.52	(0.72, 3.22)	0.270
GSF renal	0.93	(0.28, 3.09)	0.908	0.43	(0.13, 1.39)	0.158
GSF frailty	0.77	(0.37, 1.61)	0.486	1.61	(0.83, 3.12)	0.156
GSF dementia	**3.94**	**(1.14, 13.64)**	**0.030**	**2.88**	**(1.29, 6.41)**	**0.010**
GSF stroke	2.76	(0.61, 12.56)	0.190	1.90	(0.69, 5.23)	0.216
GSF others combined	0.78	(0.31, 1.92)	0.586	0.93	(0.41, 2.09)	0.854
Age	1.00	(0.98, 1.02)	0.977	1.00	(0.98, 1.01)	0.606
Co-morbidities 0/1	1.00			1.00		
2	0.53	(0.22,1.28)	0.156	0.58	(0.27, 1.26)	0.171
3+	0.64	(0.29, 1.43)	0.275	0.66	(0.33, 1.29)	0.218
Gender mMale	1.00			1.00		
Female	1.57	(0.80, 3.11)	0.193	**2.00**	**(1.09, 3.64)**	**0.024**
Living with others	1.00			1.00		
Living alone	0.61	(0.29, 1.29)	0.197	1.07	(0.56, 2.04)	0.836
Living in residential/nursing	1.33	(0.34, 5.12)	0.680	1.28	(0.46, 3.55)	0.638

## Discussion

This paper has described levels of physical, psychological and other religious, social, functional and treatment related burdens using the SPARC screening tool amongst a population of patients in two acute settings in the UK. The results show that patients meeting GSF criteria were significantly older than the remaining hospital population included in the study and that a greater proportion lived alone in the community or had been admitted from residential or nursing home care. Elevated burden across a range of items within SPARC is apparent. The results also indicate differences between those patients with (GSF group) and without palliative care need (non-GSF group) in the prevalence of burden. In particular those patients with palliative care need were more likely to identify higher levels of physical burden, including: weakness; tiredness; shortness of breath; restlessness and agitation. Patients without palliative care needs were more likely to report being bothered by pain. These findings endorse the emphasis given to these needs in relation to workforce training within recent UK guidance [23]. These data echo the findings of research already undertaken and which has addressed the physical symptoms of those with supportive care needs which suggests considerable symptom burden [24]. Patients with palliative care needs were more likely to report being bothered by a number of psychological burdens, including: anxiety; low mood; poor concentration and confusion. Together these physical and psychological findings resonate with previous evidence of the supportive care needs of those with cancer [25], stroke [26], COPD and renal failure [8] and dementia [27]. The results also demonstrate that despite meeting GSF criteria, few patients had been referred to SPC services and of those being referred almost all were suffering from malignant disorders. These findings are consistent with recent work undertaken in an Australian acute setting [17]. The findings also resonate with other studies which have looked at the palliative care needs of older people in general hospital settings who have not been referred to SPC [28].

A number of variables were shown to be significant predictors of both physical and psychological burden. Heart failure was less likely to be associated with physical outcomes, whilst dementia is a significant predictor of physical burden. These data provide further evidence of elevated levels of physical symptoms amongst dementia patients, requiring interventions of a supportive and palliative nature and adds to an existing literature [27]. Female gender was a significant predictor of psychological burden (OR 2). Whilst it is consistent with primary care studies and other evidence on common mental disorder and gender, this finding is at odds with previous evidence in relation to psychological difficulties at the end life which would suggest that the prevalence amongst male patients is higher [29]. This may, however, be an effect of the higher proportion of women identified under the GSF criteria for dementia (n = 20). The relationship between multiple conditions and poor psychological status has been demonstrated elsewhere. Studies focusing on those patients eligible for palliative forms of care are limited to the relationship between disease specific conditions, co-morbidities and psychological distress and the development of new co-morbidities is associated with readmission of palliative care patients [30]. Despite this, co-morbidity does not appear to have been a significant predictor of either physical or psychological burden in this study.

Dementia is again implicated as a significant predictor of psychological burden (OR 2.88) and adds further credence for the use of palliative regimes in providing care and treatment to address psychological need for this vulnerable patient group [31]. Furthermore, it has been demonstrated on numerous occasions that this group of patients faces a number of challenges in accessing palliative and supportive regimes of care and that these challenges are both cultural and organisational [32,33]. These data suggest that in addressing the complex physical and psychological needs of an ageing population, care teams will be increasingly required to adapt integrated palliative approaches to a cognitively frail hospital population.

### Study limitations

The study was undertaken in two large hospitals in the UK within two, two week periods during 2010. The study was designed to maximise the number of participants included but inevitably there was some exclusions which were not due to chance alone. Notable is the exclusion of those who did not consent to participate in this study (N = 705). In addition, a pragmatic decision was taken not to undertake translation or interpretation and as a result all non-English speakers were excluded from the study. Finally, those people who were unable to consent and did not have a close relative or friend who was available were also excluded. This study sought to explore the physical and psychological burden amongst a sub-population of hospital in-patients as determined by use of the GSF. Whilst the GSF is used here, its reliability in identifying patients who require palliative care is currently open to critical discussion. For example, it has not been validated as prognostic tool in terms of survival; nor does it purport to be an holistic needs assessment of palliative care need. The small sample size used in the regression analysis should also be mentioned here. The final number included in the analysis was 183 and may have contributed to a lack of power and a Type II error. Finally, dementia was identified as a predictor of both physical and psychological burden. It was noted within the methods section of this paper that where a person did not have capacity to consent to participation a personal consultee (relative or close friend) was approached and invited to participate on their behalf. As such it should be noted that data concerning those patients identified as having dementia were more likely to be reported by proxy via a consultee. Whilst every effort was made during the study to ensure validity of these data, previous evidence would suggest that such proxy reports are likely to over-estimate symptom burden to some extent [34]. This would suggest that caution should be added to the findings that a diagnosis of dementia is a diagnostic factor predicting burden.

## Conclusion

This paper has highlighted the physical, psychological, spiritual and social burden experienced by a large sample of hospital in-patients in two large acute settings in the UK. The paper draws attention to the elevated levels of burden amongst those patients identified as having palliative care needs using GSF criteria. Pain, fatigue, low mood and anxiety were particularly prevalent. Importantly the paper also draws attention to the very low level of referral to SPC services for those patients identified as requiring palliative care. Dementia is noted as a predictor of physical burden. Dementia and female gender are noted as predictors of psychological burden. The paper has drawn attention to the need to develop a workforce which is capable of meeting the palliative and supportive needs of an ageing and potentially cognitively frail hospital population in the future.

## Competing interests

In the past five years have you received reimbursements, fees, funding, or salary from an organization that may in any way gain or lose financially from the publication of this manuscript, either now or in the future? No

Do you hold any stocks or shares in an organization that may in any way gain or lose financially from the publication of this manuscript, either now or in the future? No

Do you hold or are you currently applying for any patents relating to the content of the manuscript? Have you received reimbursements, fees, funding, or salary from an organization that holds or has applied for patents relating to the content of the manuscript? No

Do you have any other financial competing interests? No

Are there any non-financial competing interests (political, personal, religious, ideological, academic, intellectual, commercial or any other) to declare in relation to this manuscript? No

The authors declare that they have no competing interests.

## Authors’ contributions

TR made a considerable contribution to the collection of the data, analysis and interpretation, drafting, revising and completion and final approval of the manuscript. CI made a considerable contribution to the design of the study, data collection, revisions and final approval of the manuscript. CG made a considerable contribution to the design of the study, data collection, revisions and final approval of the manuscript. CP made a considerable contribution to the analysis and interpretation of the data, revisions and final approval of the manuscript. MG made a considerable contribution to the design of the study, data collection, revisions and final approval of the manuscript. BN made a considerable contribution to the design of the study and final approval of the manuscript. All authors read and approved the final manuscript.

## Pre-publication history

The pre-publication history for this paper can be accessed here:

http://www.biomedcentral.com/1472-684X/12/11/prepub
